# Loss of a Branch Sugar in the Acinetobacter baumannii K3-Type Capsular Polysaccharide Due To Frameshifts in the *gtr6* Glycosyltransferase Gene Leads To Susceptibility To Phage APK37.1

**DOI:** 10.1128/spectrum.03631-22

**Published:** 2023-01-18

**Authors:** Olga Y. Timoshina, Anastasiya A. Kasimova, Mikhail M. Shneider, Nikolay P. Arbatsky, Alexander S. Shashkov, Andrey A. Shelenkov, Yuliya V. Mikhailova, Anastasiya V. Popova, Ruth M. Hall, Yuriy A. Knirel, Johanna J. Kenyon

**Affiliations:** a M. M. Shemyakin and Yu. A. Ovchinnikov Institute of Bioorganic Chemistry, Russian Academy of Sciences, Moscow, Russia; b N. D. Zelinsky Institute of Organic Chemistry, Russian Academy of Sciences, Moscow, Russia; c Central Scientific Research Institute of Epidemiology, Moscow, Russia; d State Research Center for Applied Microbiology and Biotechnology, Obolensk, Russia; e School of Life and Environmental Science, University of Sydney, Sydney, Australia; f Centre for Immunology and Infection Control, School of Biomedical Sciences, Faculty of Health, Queensland University of Technology, Brisbane, Australia; University of Pittsburgh School of Medicine

**Keywords:** *Acinetobacter baumannii*, capsular polysaccharide, K locus, KL3, K3-v1, APK37.1

## Abstract

The type of capsular polysaccharide (CPS) on the cell surface of Acinetobacter baumannii can determine the specificity of lytic bacteriophage under consideration for therapeutic use. Here, we report the isolation of a phage on an extensively antibiotic resistant ST2 A. baumannii isolate AB5001 that carries the KL3 CPS biosynthesis gene cluster predicting a K3-type CPS. As the phage did not infect isolates carrying KL3 or KL22 and known to produce K3 CPS, the structure of the CPS isolated from A. baumannii AB5001 was determined. AB5001 produced a variant CPS form, K3-v1, that lacks the β-d-Glс*p*NAc side chain attached to the d-Gal*p* residue in the K3 structure. Inspection of the KL3 sequence in the genomes of AB5001 and other phage-susceptible isolates with a KL3 locus revealed single-base deletions in *gtr6*, causing loss of the Gtr6 glycosyltransferase that adds the missing d-Glс*p*NAc side chain to the K3 CPS. Hence, the presence of this sugar profoundly restricts the ability of the phage to digest the CPS. The 41-kb linear double-stranded DNA (dsDNA) phage genome was identical to the genome of a phage isolated on a K37-producing isolate and thus was named APK37.1. APK37.1 also infected isolates carrying KL116. Consistent with this, K3-v1 resembles the K37 and K116 structures. APK37.1 is a *Friunavirus* belonging to the *Autographiviridae* family. The phage-encoded tail spike depolymerase DpoAPK37.1 was not closely related to Dpo encoded by other sequenced *Friunaviruses*, including APK37 and APK116.

**IMPORTANCE** Lytic bacteriophage have potential for the treatment of otherwise untreatable extensively antibiotic-resistant bacteria. For Acinetobacter baumannii, most phage exhibit specificity for the type of capsular polysaccharide (CPS) produced on the cell surface. However, resistance can arise via mutations in CPS genes that abolish this phage receptor. Here, we show that single-base deletions in a CPS gene result in alteration of the final structure rather than deletion of the capsule layer and hence affect the ability of a newly reported podophage to infect strains producing the K3 CPS.

## INTRODUCTION

On a global scale, Acinetobacter baumannii has been listed as one of the six leading bacterial pathogens responsible for the majority of deaths associated with antimicrobial resistance (1). In recent years, lytic bacteriophage (phage) that selectively infect and lyse bacteria have been reinvestigated for use in treating otherwise untreatable multidrug-resistant infections and have shown promise as therapeutic alternatives or adjuncts to antibiotic chemotherapy (2–5). In most cases, the initial receptor involved in phage adsorption to the A. baumannii cell surface is the capsular polysaccharide (CPS) layer surrounding the bacterial cell (6–9). However, individual strains produce just one of a large number of CPS types found in the species. Hence, screening for phage activity against diverse collections of A. baumannii strains that differ in the type of CPS produced is needed to build versatile phage libraries or biobanks for a more targeted approach to therapeutics screening.

CPS type is largely determined by genes found at the chromosomal K locus (KL) for CPS biosynthesis in A. baumannii genomes (10), and more than 240 different sets of genes have been identified at this location to date (11–13). However, the CPS structures for only ~75 different KL have been determined. Differences between known structures include differences in the types of carbohydrate constituents and noncarbohydrate decorations present in the repeating oligosaccharide (OS) K-units that make up the CPS as well as the anomeric configurations of the glycosidic linkages between sugars and also between K-units (14–22). As more and more CPS structural data have become available, prediction of CPS structures from whole-genome sequences via detection of specific sets of CPS biosynthesis genes at the K locus has become possible using the bioinformatics typing tool Kaptive (11, 13).

**FIG 1:**
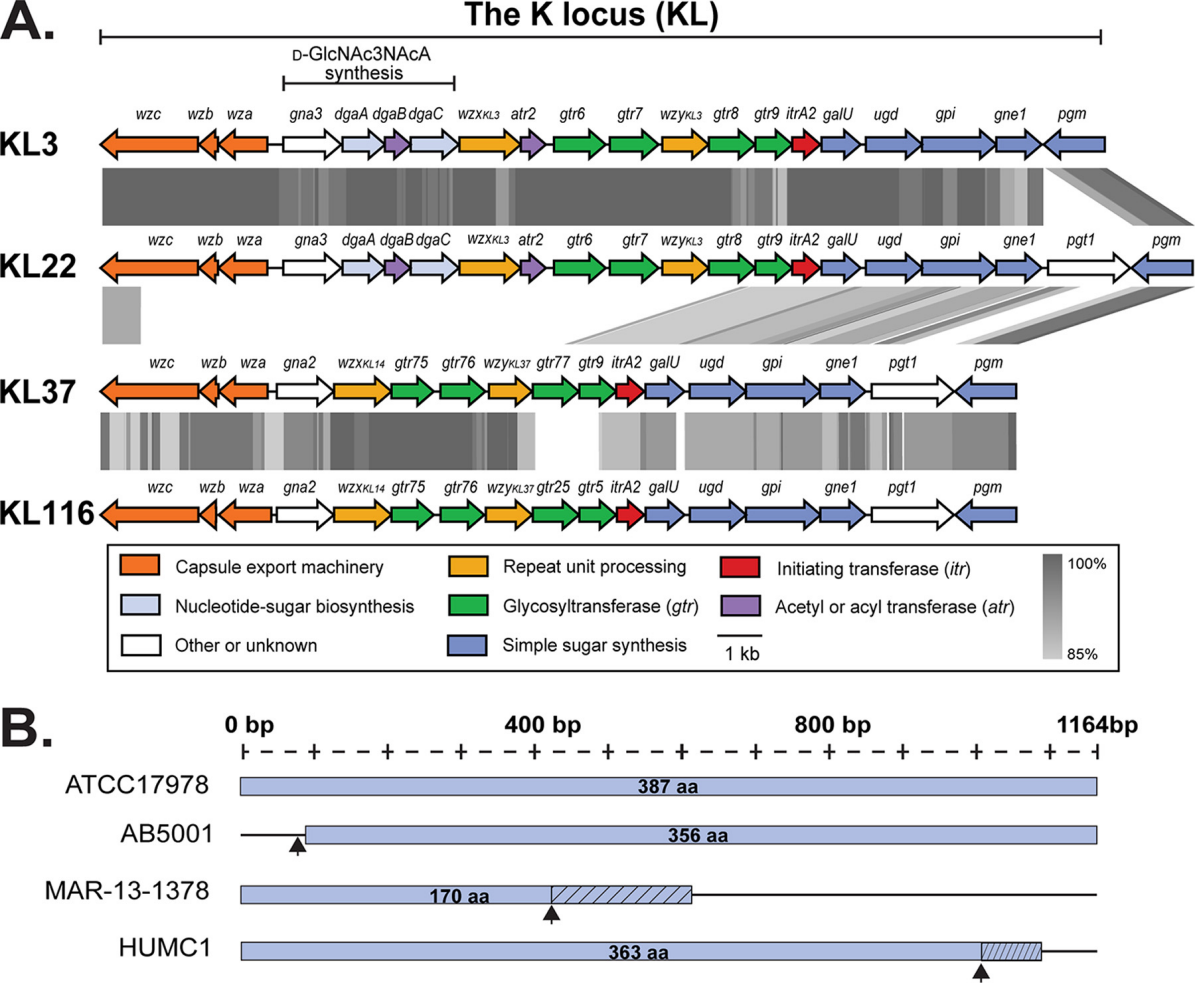
(A) Comparison of the A. baumannii KL3, KL22, KL37, and KL116 CPS biosynthesis gene clusters. Figures were drawn using EasyFig (44) with K locus sequences from NCBI accession numbers CP012004.1 (KL3, ATCC 17978), KC526920.1 (KL22, LUH5537), KX712115.1 (KL37, UV_1036), and CP020590 (KL116, 15A34). The scale bar is shown below. Predicted functional categories of gene products are indicated by colors (legend shown below), and gray shading is protein sequence identity determined by tblastx with the scale also shown below. (B) Positions of the deletions or insertions in the *gtr6* gene and length of predicted gene products. Length of the gene is shown above, and length of gene product (blue box) is shown. Black arrows indicate the locations of the single-base deletion in AB5001/AB4932/AB4957 and MAR-13-1378 or the IS*Aba*13 insertion sequence in KL22 of HUMC1 (25). Hashed portion in MAR-13-1378 and HUMC1 products indicate extension past the deletion/insertion to the nearest alternate stop codon.

Treatment using alternate phage therapies is most appropriate for infections that fail to respond to clinically available antibiotics, and such strains often carry either KL3 or KL22 CPS biosynthesis gene clusters. In a recent analysis of publicly available A. baumannii genome assemblies, ~20% of sequenced isolates were found to carry KL3 or KL22 (11), which differ only in the presence of a single gene (annotated as *pgt1* in Fig. 1A) that has no known role in CPS biosynthesis (Fig. 1A). The differences between these KL therefore do not affect the carbohydrate composition of the CPS produced (23–26), and the shared structure is referred to as the K3 type (11). This CPS type has been associated with increased virulence (27, 28) and also with carbapenem resistance in nosocomial cases of bacteremia and pneumonia and increased rates of admission to intensive care units (29). Hence, there is a need for phage specific for K3.

**FIG 2:**
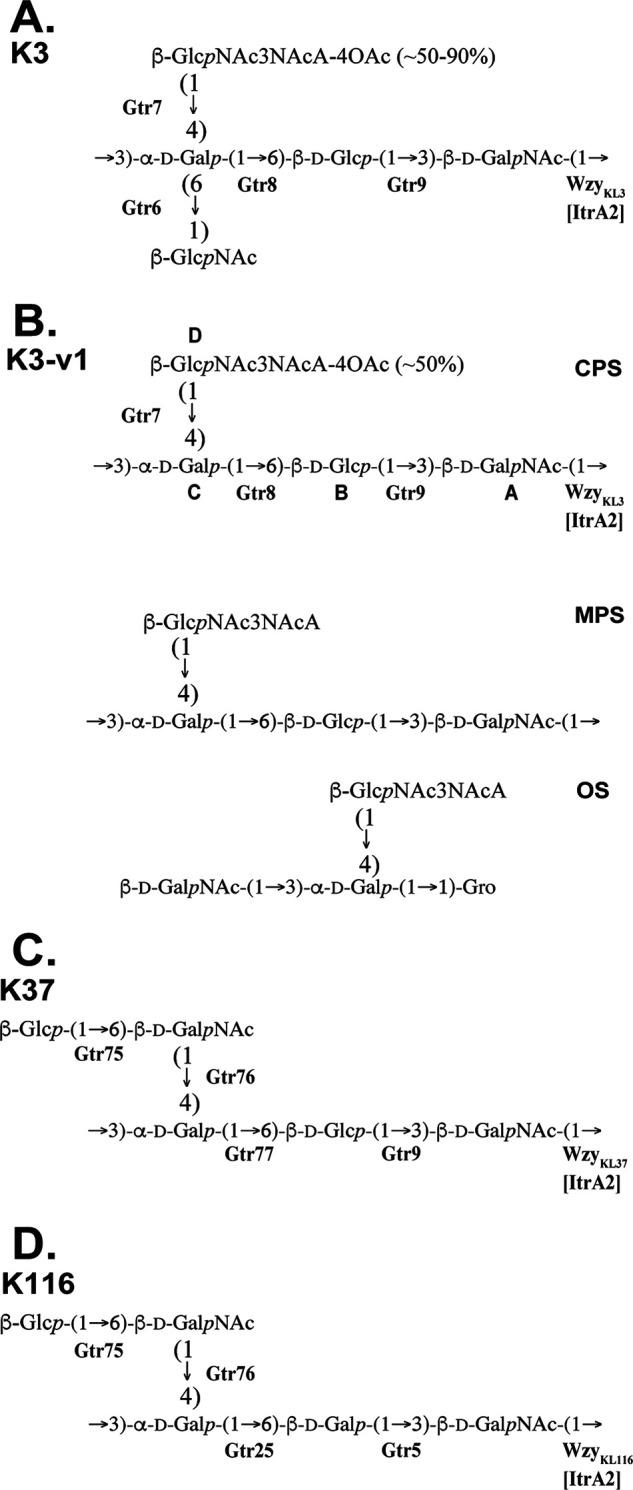
CPS structures from A. baumannii isolates. (A) K3-type from isolates with KL3 (ATCC 17978 [23] and SMAL [30]) or KL22 (15827 [25], LUH5537 [24], and SK44 [26]). The degree of *O*-acetylation of d-GlcNAc3NAcA is ~50% in KL3 strains and ~90% in KL22 strains. (B) K3-v1 CPS type and MPS and OS from A. baumannii AB5001. The degree of *O*-acetylation of d-GlcNAc3NAcA is ~50%. (C) K37 CPS (24, 38). (D) K116 CPS (38). Glycosyltransferase, Itr, and Wzy names are shown next to the linkage each is assigned to.

The K3 structure includes a main chain comprised of a →3)-α-d-Gal*p*-(1→6)-β-d-Glс*p*-(1→3)-β-d-Gal*p*NAc-(1→ trisaccharide, with two different sugars, 2,3-diacetamido-2,3-dideoxy-d-glucuronic acid (d-GlcNAc3NAcA) and a d-Glc*p*NAc branching from the d-Gal*p* residue at positions C4 and C6, respectively (Fig. 2A) (23–26, 30). Recently, a study showed that the loss of the branching d-Glc*p*NAc sugar in A. baumannii isolate HUMC1 carrying KL22 via interruption of the gene for the Gtr6 glycosyltransferase by an IS*Aba*13 insertion sequence significantly increased virulence of cells by inhibiting phagocytosis *in vivo* (25). Although the loss of this sugar appears clinically important, the effect of this structural variation on phage susceptibility has not been explored.

In this study, we report the properties of a phage isolated on an A. baumannii isolate carrying the KL3 locus and thus predicted to produce a K3 CPS. The phage did not grow on known K3 producers, and the reason for this was traced to an alteration in the CPS structure caused by single-base deletions in the *gtr6* gene that alter the final structure.

## RESULTS

### A. baumannii AB5001 is an extensively antibiotic-resistant GC2 isolate carrying KL3.

A. baumannii isolate AB5001 was recovered from a blood infection of a US military patient in 2008 (31). Using Kaptive (11), the available draft genome sequence (NCBI accession number LREN00000000.1) (32) was found to include the KL3 locus and hence predicted to produce a K3-type CPS. The genome belongs to ST2 in the A. baumannii Institut Pasteur multilocus sequence typing (MLST) scheme and ST452 in the Oxford MLST scheme (Table 1), identifying it as a strain belonging to the major globally disseminated clonal lineage, Global Clone 2 (GC2), which includes pan-resistant strains for which bacteriophage therapy is warranted. AB5001 is recorded as resistant to most agents currently used for therapy, including imipenem, broad-spectrum cephalosporins, aminoglycosides, amikacin and gentamicin, sulfonamides, tetracycline, and fluoroquinolones (see Table S1 in reference 31). Consistent with this phenotype, from the draft genome sequence, it was found that AB5001 carries the *oxa23* gene for resistance to carbapenems and *bla*_PER-1_, which confers resistance to ceftazidime, cefotaxime, and aztreonam. AB5001 also carries the *aphA1* (kanamycin and neomycin resistance) gene and the *aphA6* (amikacin resistance) and *aacC1* (gentamicin resistance) genes. The *sul2* sulfonamide resistance, *tet*(B) tetracycline resistance, and *strAB* streptomycin resistance genes are also present.

**TABLE 1:** A. baumannii strains tested in this study

Strain name	Country	Yr	Source	ST^ip^^*h*^	ST^OX^^*i*^	K locus	Resistance determinants	NCBI accession no.
ATCC 17978^*a*^	France	1951	Meninges	ST437	ST112	KL3	*sul2*	CP012004.1
ATCC 19606	USA	<1948	Urine	ST52	ST931	KL3	*sul2*	CP045110.1
AB5711 (MRSN1310)	USA	2009	Blood/sepsis	ST2	ST452	KL3	*aacC1*, *aphA6*, *bla*_PER-1_, *oxa23* (x3), *strA* (x2), *strB*, *sul2*	AHAJ00000000.1
AB5001 (MRSN954)	USA	2008	Blood/sepsis	ST2	ST452	KL3	*aacC1*, *aphA1*, *aphA6*, *bla*_PER-1_, *bla*_TEM-116_, *oxa23*, *strA*, *strB*, *sul2*	LREN00000000.1
AB4932 (MRSN949)	USA	2008	Sputum	ST2	ST452	KL3	*aacC1*, *aphA6*, *bla*_PER-1_, *oxa23*, *strB*, *sul2*	LREK00000000.1
AB4957 (MRSN951)	USA	2008	Sacral/osteomyelitis	ST2	ST452	KL3	*aacC1*, *aphA6*, *bla*_PER-1_, *oxa23*, *strB*, *sul2*	LREL00000000.1
MAR 13-1378	Russia	2013	Respiratory tract	ST78	New^*b*^	KL3	ND^*c*^	JAPQKD000000000
LUH5537^*e*^	Netherlands	Unknown	Unknown	ST2	ST1839	KL22	*aacC1*, *aadA1*, *aphA1*, *bla*_TEM-1D_, *catA1*, *qacE*, *sul1*	KC526920.1 ^*d*^
RES-1687	Russia	2003	Skin and soft tissue	ND^*c*^	ND^*c*^	KL22	*aadA24*, *aadB*, *aphA6*, *sul2*	JAPQKA000000000
REV-3116	Russia	2007	Blood	ST2	ST281	KL22	*aacC1*, *aadA1*, *aphA1*, *bla*_TEM-1D_, *qacE*, *sul1*	JAPQKC000000000
NIPH 146^*f*^	Czech Republic	1993	Wound	ST25	ST276	KL37		APOU00000000.1
KZ-1101	Kazakhstan	2016	Skin/soft tissue	ST132	ST2213	KL37		JAPYKX000000000.1
MAR-303^*g*^	Russia	2011	Inpatient	ST12	ST953	KL116	*aadB*, *aphA6*, *bla*_OXA-120_	JANSJS000000000
TP1	USA	2016	Peritoneal drain	ST570	ST1578	KL116	*aac(6′)-Ib-cr*, *aacA4*, *aadA1*, *aphA1*, *armA*, *bla*_TEM-1D,_ *catB8*, *mph*(E), *msr*(E), *oxa23*, *qacE*, *sul1*	NZ_CP056784.1
TP3	USA	2016	Pancreatic drain	ST570	ST1578	KL116	*aacA4*, *aadA1*, *aphA1 (x2)*, *aphA6*, *armA*, *bla*_NDM-1,_ *bla*_TEM-1D,_ *catB8*, *mph*(E), *msr*(E), *oxa23*, *qacE*, *sul1*	NZ_CP060013.1

a The structure of CPS produced by this strain was determined previously (23, 25). CPS includes the d-GlcNAc branch.

b Single locus variant of ST1961 and ST2331.

c ND, not detected.

d Only K locus sequence is available.

e The structure of CPS produced by this strain was determined previously (24). CPS includes the d-GlcNAc branch.

f The structure of CPS produced by this strain was determined previously (24, 38).

g The structure of CPS produced by this strain was determined previously (38).

h For ST^ip^: sequence type in Institut Pasteur scheme.

i For ST^OX^: sequence type in Oxford scheme.

### Isolation of a new A. baumannii bacteriophage.

**FIG 3:**
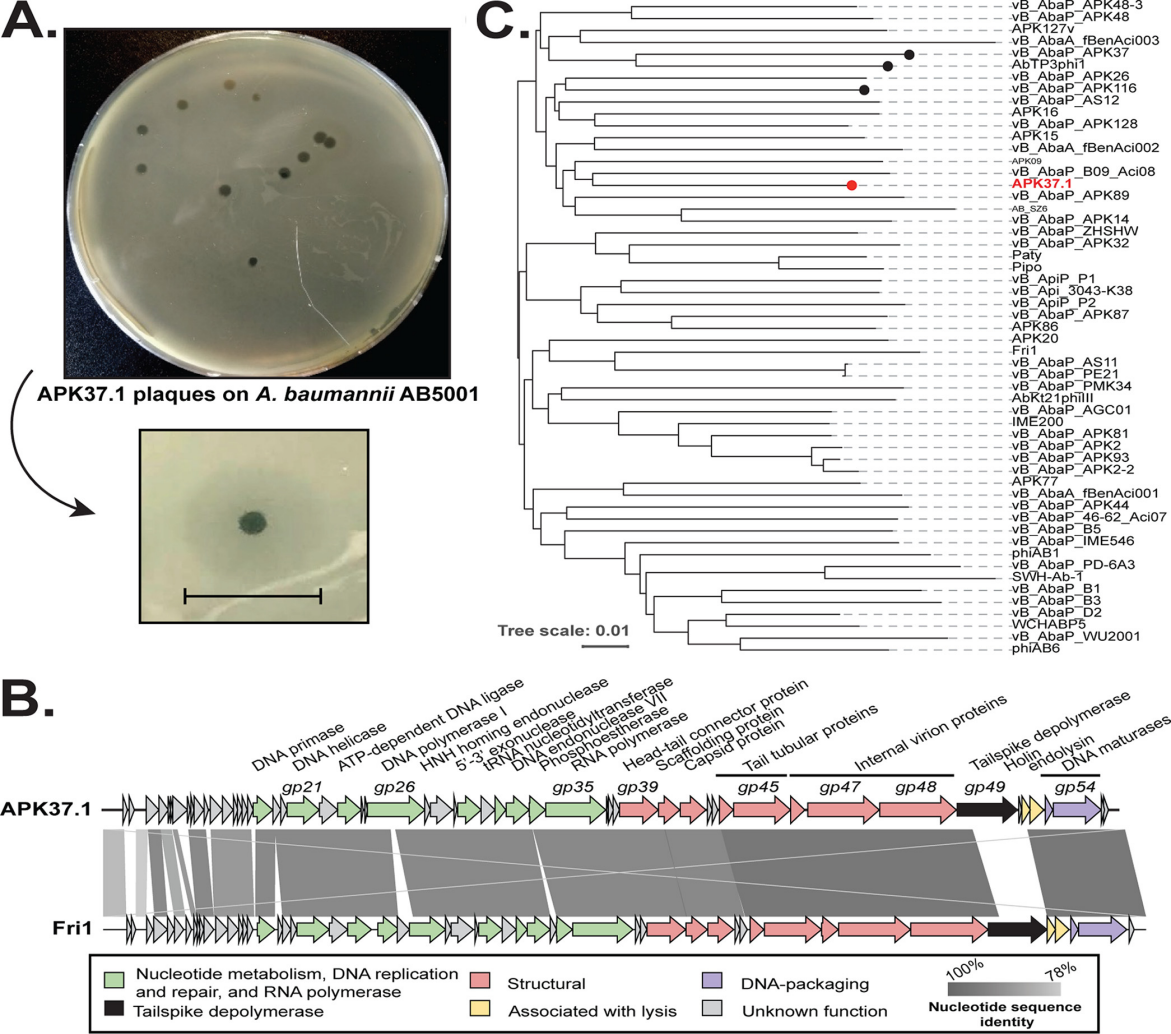
Characterization of APK37.1. (A) Plaques with opaque haloes formed by phage APK37.1 on a lawn of A. baumannii AB5001. A magnified image of a plaque is shown below, with the bar indicating halo diameter. (B) Pairwise nucleotide sequence comparison of APK37.1 and Fri1 genomes. Shading shows DNA sequence identity, and the scale is shown below. Open reading frames are colored by functional category with the key shown below. The figure was constructed by Easyfig (44). (C) Proteomic phylogeny of related *Friunavirus* genomes. Circles on node tips indicate phage that have demonstrated specificity for strains that carry the KL37 or KL116 CPS biosynthesis gene clusters. Red denotes phage APK37.1.

Samples of sewage water collected in 2018 from the Moscow region in Russia were used to isolate phage that could infect the extensively antibiotic-resistant A. baumannii isolate AB5001 carrying KL3. A phage producing relatively large clear plaques (about 3 to 4 mm in diameter) surrounded by visible halos (Fig. 3A) was isolated on a lawn of AB5001. The phage was recovered, and the nucleotide sequence of the genome was determined. The phage genome sequence was identical to that of a phage independently isolated from the same sewage sample on a lawn of A. baumannii isolate KZ-1101 that carries the KL37 CPS biosynthesis gene cluster (Fig. 1A). Hence, AB5001 and KZ-1101 were infected by one and the same phage, which, following the nomenclature used by Popova and colleagues (9), was assigned the name APK37.1.

### Phage APK37.1.

The APK37.1 genome was annotated and submitted to GenBank under accession number MZ967493.1. APK37.1 has a linear double-stranded DNA (dsDNA) genome of 40,966 bp with terminal repeats of 399 bp and an overall G+C content of 39.2%, consistent with other phage specific to A. baumannii (9). A total of 56 predicted open reading frames (ORFs) all oriented in the same direction were found (Fig. 3B), with one ORF, named gp35, predicted to encode a DNA-dependent RNA polymerase (GenPept accession number UAW07714.1), which is characteristic of members of the phage family *Autographiviridae* (33). Genome comparison of APK37.1 through calculation of average nucleotide identity (ANI) revealed an ANI of 92.76% with the prototype podophage Fri1 (GenBank accession number NC_028848.1), which belongs to *Friunavirus* of the *Beijerinckvirinae* subfamily within the family *Autographiviridae* (34). A pairwise sequence comparison of APK37.1 and Fri1 revealed significant sequence homology (Fig. 3B), particularly in the “late genome” region that includes genes for common podophage structural proteins (*gp39-gp48* in APK37.1). A proteomic tree calculated from the nucleotide sequences of related A. baumannii podophage in the *Friunavirus* genus (Fig. 3C) revealed that APK37.1 groups among members of this family but in a different clade to Fri1. Taken together, the data indicated that APK37.1 belongs to the *Friunavirus* genus in the *Autographiviridae* family.

### Phage APK37.1 cannot lyse other K3-producing strains.

To confirm the specificity of APK37.1 for K3 CPS, the phage was applied to a small collection of 10 other isolates (Table 1) with whole-genome sequences that were known or were predicted to include either KL3 (*n* = 6) or KL22 (*n* = 4) sequences at the K locus. These strains included ATCC 17978 (KL3) and LUH5537 (KL22), for which the chemical structure of the CPS had been determined previously and shown to be the K3 type (23–25). However, APK37.1 did not infect these two strains. In addition to AB5001, phage APK37.1 could infect only three other isolates that carried KL3 (Table 2). Hence, overall, three KL3 isolates (43%) and all three KL22 isolates (100%) were found to be resistant to APK37.1 infection. The difference in APK37.1 infectivity suggested that AB5001 might produce a different or modified CPS structure, and, as the chemical structures of CPS produced by the isolates susceptible to APK37.1 infection have not been previously elucidated, AB5001 was chosen for further chemical analyses by NMR.

**TABLE 2:** Susceptibility of A. baumannii isolates with KL3 or KL22 to APK37.1 infection

Strain name	K locus	Susceptibility to APK37.1	*gtr6* frameshift	Frameshift details	First base position
ATCC 17978	KL3	−	−	−	−
ATCC 19606	KL3	−	−	−	−
AB5711	KL3	−	−	−	−
LUH5537	KL22	−	−	−	−
RES-1687	KL22	−	−	−	−
REV-3116	KL22	−	−	−	−
AB5001	KL3	+	+	−A in string of 7 × A	81
AB4932	KL3	+	+	−A in string of 7 × A	81
AB4957	KL3	+	+	−A in string of 7 × A	81
MAR 13-1378	KL3	+	+	−A in string of 9 × A	440

### Structural elucidation of the AB5001 CPS.

A high-molecular-mass CPS was isolated from cells of A. baumannii AB5001 by the phenol-water procedure (35). Sugar analysis of the CPS showed the presence of Glc, Gal, and GalN in a ratio of 1.0 to 0.5 to 1.0, respectively.

The CPS was studied by nuclear magnetic resonance (NMR) spectroscopy, including one-dimensional ^1^H and ^13^C NMR experiments and two-dimensional ^1^H,^1^H correlation spectroscopy (COSY), total correlation spectroscopy (TOCSY), rotating-frame nuclear Overhauser effect spectroscopy (ROESY) (Fig. S1 in the supplemental material), ^1^H,^13^C heteronuclear single quantum coherence (HSQC) (Fig. S2), and heteronuclear multiple-bond correlation (HMBC) experiments. Five sugar spin systems, including those for α-Gal (unit **A**), β-Glc (unit **B**), β-GalNAc (unit **C**), β-GlcNAc3NAcA, and β-GlcNAc3NAcA-4Ac (unit **D′**) were identified (Table 3), all monosaccharides being in the pyranose form.

**TABLE 3:** ^1^H and ^13^C NMR chemical shifts (δ, ppm) of the CPS of A. baumannii AB5001 and MPS derived from *O*-deacetylation of the CPS

Sugar residue	C-1 *H-1*^*a*^	C-2 *H-2*	C-3 *H-3*	C-4 *H-4*	C-5 *H-5*	C-6 *H-6 (6a,6b)*
CPS^*b*^						
→3)-α-d-Gal*p*-(1→ A	99.7	66.8	81.6	77.0	71.4	61.7
	*4.94*	*3.73*	*3.92*	*4.40*	*3.64*	*3.65*, *3.79*
→6)-β-d-Glc*p*-(1→ B	106.0	74.4	77.1	70.3	77.6	66.6
	*4.56*	*3.32*	*3.48*	*3.60*	*3.59*	*3.73*, *4.01*
→3)-β-d-Gal*p*NAc-(1→ C	105.0	52.9	82.0	71.4	76.0	62.5
	*4.65*	*4.14*	*3.87*	*3.93*	*3.71*	*3.82*, *3.82*
β-d-Glc*p*NAc3NacA-(1→ D	102.6	54.8	56.2	71.4	75.0	174.1
	*5.08*	*3.84*	*4.03*	*3.64*	*4.03*	
β-d-Glc*p*NAc3Nac4AcA-(1→ D′	102.4	54.4	54.2	72.1	77.1	174.1
	*5.08*	*3.96*	*4.23*	*4.96*	*3.90*	
MPS^*c*^						
→3)-α-d-Gal*p*-(1→ A	100.0	69.1	81.8	77.4	71.8	62.1
	*4.93*	*3.73*	*3.92*	*3.40*	*3.93*	*3.65*, *3.77*
→6)-β-d-Glc*p*-(1→ B	106.2	74.7	77.5	70.7	75.9	67.1
	*4.54*	*3.31*	*3.48*	*3.59*	*3.59*	*3.75*, *4.00*
→3)-β-d-Gal*p*NAc-(1→ C	105.3	53.2	82.2	69.5	76.3	62.8
	*4.66*	*4.13*	*3.88*	*4.16*	*3.71*	*3.82*, *3.82*
β-d-Glc*p*NAcA-(1→ D	102.9	55.1	56.5	71.6	77.1	175.2
	*5.10*	*3.85*	*4.03*	*3.67*	*3.97*	
OS^*d*^ →3)-α-d-Gal*p*-(1→ A	100.1	68.8	81.7	77.1	71.8	61.8
	*4.88*	*3.71*	*3.92*	*4.39*	*3.93*	*3.65*, *3.77*
→3)-β-d-Gal*p*NA*c*-(1→ C	105.4	54.2	72.6	69.1	76.3	62.6
	*4.57*	*4.00*	*3.72*	*3.96*	*3.69*	*3.83*
β-d-Glc*p*NAcA-(1→ D	102.6	54.9	56.0	71.6	77.2	175.9
	*5.08*	*3.82*	*4.04*	*3.62*	*3.88*	
→1)-Gro B′	70.1	71.4	63.9			
	*3.56*, *3.73*	*3.91*	*3.62*, *3.66*			

a H NMR chemical shifts are italicized.

b Chemical shifts for the *N*-acetyl group are δ_H_ 1.90 to 2.07, δ_C_ 22.2 to 23.8 (Me), and 175.8 to 176.0 (CO).

c Chemical shifts for the *N*-acetyl group are δ_H_ 2.00 to 2.01, δ_C_ 23.4 to 23.7 (Me), and 176.1 to 176.3 (CO).

d Chemical shifts for the *N*-acetyl group are δ_H_ 1.99 to 2.01, δ_C_ 23.7 to 24.1 (Me), and 176.0 to 176.1 (CO).

In the ^1^H NMR spectrum, there were five signals for anomeric protons at δ 4.56 to 5.08, five signals of H-2 and H-3 of the NAc-linked carbons at δ 3.87 to 4.23 and H-4 of unit **D′** at an OAc-linked carbon at δ 4.96. The ^13^C NMR spectrum showed five signals for anomeric carbons at δ 99.7 to 106.0, signals of N-linked C-2 and C-3 of units **C**, **D**, and **D′** at δ 52.9 to 54.8, and a signal of a CO_2_H group (C-6) of units **D** and **D′** at δ 174.1.

The ^1^Н and ^13^С NMR spectra of the CPS were assigned using two-dimensional ^1^Н,^1^Н COSY, ^1^Н,^1^Н TOCSY, and ^1^Н,^13^C HSQC (Fig. S2) experiments. The positions of substitution and the sequence of the monosaccharide residues were established by ^1^Н,^1^Н ROESY (Fig. S1) and ^1^Н,^13^C HMBC experiments, which showed correlations between atoms of the neighboring monosaccharide residues.

In the ^1^Н,^1^Н TOCSY spectrum, there were correlations H-1/H-2–H-4 for the sugars having the galacto configuration (units **A** and **C**) and H-1/H-2–H-6 for the sugars having the gluco configurations (units **B**, **D**, and **D′**). Units **D** and **D′** were identified as hexuronic acid derivatives based on the correlation between CO_2_H (C-6) and H-5 at δ_C/_δ_H_ 174.1/4.03 and 174.1/3.90, respectively, in the ^1^H,^13^С HMBC spectrum.

Low-field positions at δ 81.6, 82.0, and 66.6 of the signals for C-3 of units **A** and **C** and C-6 of unit **B**, respectively, showed that the CPS is branched, with three monosaccharide residues (**A** to **C**) in the main chain and unit **D** attached as a side chain.

*O*-Deacetylation was performed to confirm the structure of the CPS shown in Fig. 2B. The modified polysaccharide (MPS) obtained was subjected to Smith degradation, which cleaved the α-d-Gal*p*-(1→6)-β-d-Glс*p* linkage to give an oligosaccharide (OS), namely, a trisaccharide glycoside with glycerol (Gro) as aglycon. The structures of the MPS and OS (Fig. 2B) were established by NMR spectroscopy as described above for the CPS (for the ^1^H,^13^C HSQC spectra, see Fig. S3 and S4; for the assigned ^1^H and ^13^C chemical shifts, see Table 3), and the CPS structure was confirmed.

Therefore, the AB5001 CPS includes a trisaccharide backbone of →3)-α-d-Gal*p*-(1→6)-β-d-Glс*p*-(1→3)-β-d-Gal*p*NAc-(1→ and a 4-*O*-acetylated d-Glc*p*NAc3NAcA residue branching from position 4 of the d-Gal*p* in the main chain. The configuration is very similar to that of the K3-type CPS. However, the second side chain, d-Glс*p*NAc, attached to position 6 of the d-Gal*p* residue is missing (Fig. 2B). Hence, the variant CPS was designated the name K3-v1.

### CPS biosynthesis gene cluster in the genome sequence of AB5001.

To determine the reason for this altered phenotype, the sequence at the K locus was reexamined. The CPS biosynthesis gene cluster in the AB5001 whole-genome sequence shares 100% coverage and 98.15% nucleotide sequence identity with KL3 from ATCC 17978 reference sequence (GenBank accession number CP018664.1), which produces the K3 CPS with the d-Glс*p*NAc side branch (23). All 20 of the expected coding sequences for KL3 were present. However, a length discrepancy of −1 bp was detected between the sequences using the Kaptive typing tool. A pairwise sequence alignment of the KL3 gene clusters from AB5001 and ATCC 17978 identified a single missing “A” residue in the *gtr6* gene in a run of seven A residues beginning 81 bases downstream from the start codon (Fig. S5). Translation from the next available start codon would therefore produce a protein of 356 amino acids (aa) (Fig. 1B), as opposed to the 387-aa wild-type protein in ATCC 17978. The Gtr6 glycosyltransferase is known to be responsible for linking the d-Glc*p*NAc branched sugar (25), and this explains the absence of this sugar in the AB5001 K3-v1 CPS.

### Frameshifts in *gtr6* determine susceptibility to infection by phage APK37.1.

Inspection of the *gtr6* sequence in the collection of KL3 and KL22 isolates used to examine the specificity of APK37.1 revealed that all six resistant isolates carried an intact copy of the *gtr6* gene (Table 2). For two other isolates, AB4932 and AB4957, that were susceptible to APK37.1 infection, the same single-base deletion at position 81 was detected in the genome. However, a different single base deletion was detected in the *gtr6* sequence from the A. baumannii isolate MAR 13-1378. This deletion is in a string of nine “A” residues beginning at position 440 from the *gtr6* start codon (Fig. S5). This frameshift leads to a truncated product with only the first 149 aa the same as the complete Gtr6 sequence (Fig. 1B). Hence, it appears that the single-base deletion near base 81 or base 440 has abrogated the function of Gtr6, leading to loss of the side chain.

### Specificity of APK37.1 for other A. baumannii CPS types.

Like other Acinetobacter podophages, APK37.1 demonstrates specificity for the type of CPS produced by A. baumannii strains infected. This is known to be directed by a depolymerase enzyme surrounding the phage tail that recognizes specific CPS structures and hydrolyses a bond in the polysaccharide (6). Visible halos in APK37.1 plaques on an AB5001 lawn (Fig. 3A) suggest depolymerase activity (34, 36), and, consistent with this, a pectate lyase fold (Pfam PF12708) characteristic of tail spike/structural depolymerases was identified in the gp49 protein sequence (black ORF in Fig. 3B; GenPept accession number UAW07728.1) encoded by the APK37.1 genome.

**FIG 4:**
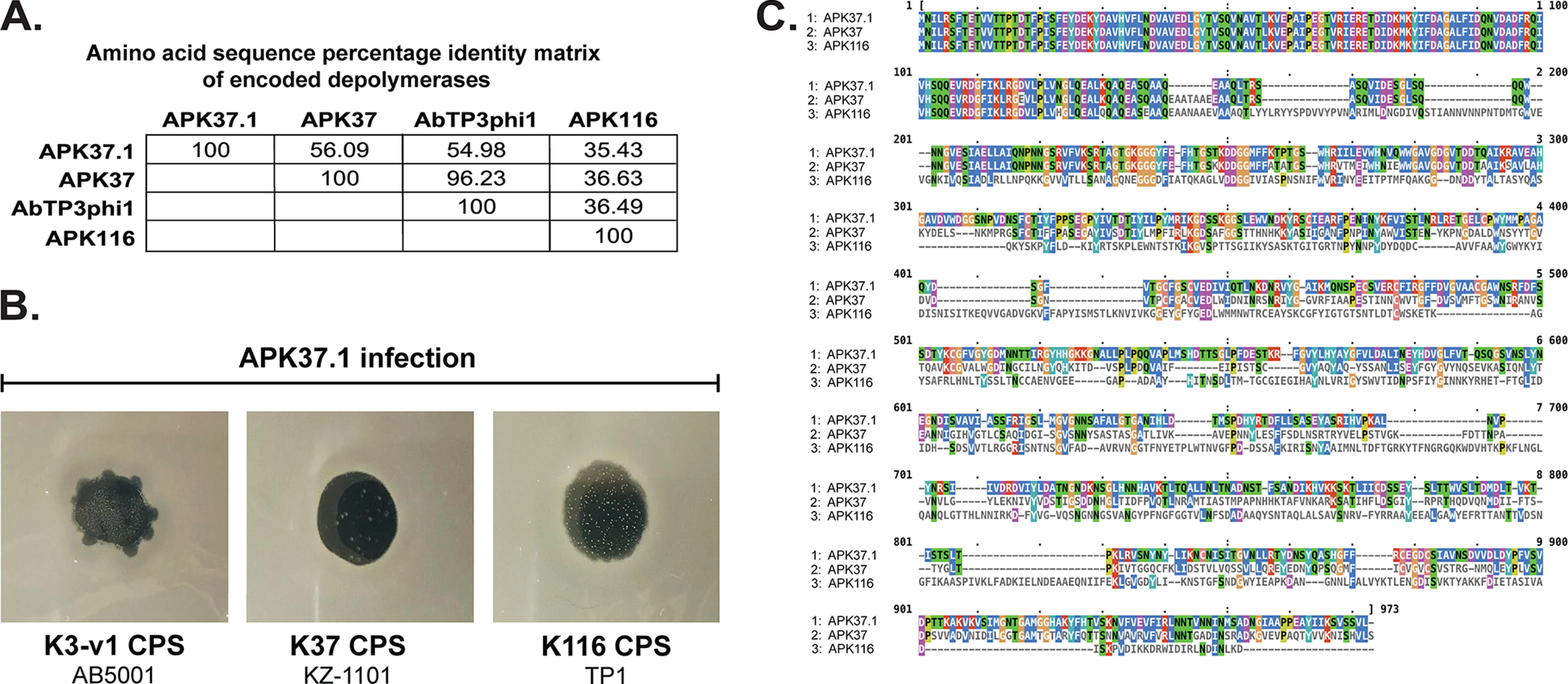
Specificity of APK37.1 for more than one CPS type. (A) Amino acid sequence percentage identity matrix of depolymerases encoded by APK37.1, APK37, AbTP3phi1, and APK116 (GenPept accession numbers UAW07728.1, AZU99445.1, UNI74976.1, and QHS01530.1, respectively). (B) APK37.1 spots on lawns of K3-v1, K37, and K116 strains. (C) Amino acid sequence alignment of depolymerases encoded by APK37.1, APK37, and AbTP3phi1. Numbers indicate amino acid ranges. Alignment is colored by identity using the CLUSTAL colormap visualized in MView.

Further inspection of the predicted product of *gp49* (here referred to as DpoAPK37.1) revealed that the amino acid sequence shares 52 to 53% amino acid sequence identity (>97% sequence coverage) with the depolymerases encoded by APK37 (gp48; GenBank accession number AZU99445) and AbTP3phi1 (gp48; GenBank accession number UNI74976.1) (Fig. 4A). APK37 and AbTP3phi1 are two other A. baumannii podophage previously shown to infect the K37 CPS-producing A. baumannii isolate NIPH 146 (9) and K116 CPS-producing A. baumannii isolate TP1 (37), respectively, and their encoded depolymerases are 96.2% identical (Fig. 4A). Considering the relationship between depolymerases and the fact that APK37.1 had also been independently isolated on the K37-producing isolate KZ-1101 (see above), the infectivity of APK37.1 on a broader set of strains carrying either KL37 or KL116 was also tested (Table 1).

APK37.1 was found to infect all five tested strains that carry either KL37 or KL116 (see Fig. 4B for APK37.1 infection of representative K37- and K116-producing isolates), indicating broad host specificity for different CPS types. Inspection of the phylogeny (Fig. 3A) revealed that APK37.1 belongs to a subclade that is separate from APK37, AbTP3phi1, and one further phage, APK116, that is also known to infect K116 CPS-producing A. baumannii strains (9). However, DpoAPK37.1 shares only 35.4% amino acid identity to the depolymerase from APK116 (Fig. 4A) and is therefore more closely related to the depolymerases from APK37 and AbTP3phi1. Similar to other capsular depolymerases encoded by members of the *Friunavirus* genus (6, 9), much of the homology between DpoAPK37.1 and depolymerases from APK37 and APK116 is at the N terminus in the first 140 amino acids (Fig. 4C), although APK37.1 and APK37 do share further homology extending up to amino acid position 250. However, the C-terminal sequence of all three depolymerases is divergent. As the C terminus is responsible for receptor recognition, diversity in this sequence may reflect differences in the range of specificity for different CPS types. However, this was not further investigated.

## DISCUSSION

In this study, we describe APK37.1, a novel A. baumannii bacteriophage of the *Friunavirus* genus, and show that it can infect only a subset of isolates that carry the KL3 or KL22 CPS biosynthesis loci. The reason for this was traced to the loss of the d-Glс*p*NAc side branch in the K3-type structure arising from a single-base deletion in the *gtr6* gene. While small changes in the nucleotide sequences of KL have previously been shown to alter phage susceptibility in some isolates (7, 37), in many cases, these changes have, or likely have, involved loss of the CPS, rendering cells resistant to further infection due to removal of the primary phage receptor. To the best of our knowledge, this study is the first report of a single-base deletion in an A. baumannii CPS biosynthesis locus leading to a demonstrated change in the composition of the structure rather than complete deletion of the CPS layer on the cell surface.

Before this study, loss of the d-Glc*p*NAc side branch in the K3 CPS had been described for the extensively resistant A. baumannii clinical isolate HUMC1 (25), in which an IS*Aba*13 sequence was found to interrupt the *gtr6* gene. The same phenotype is produced by two different and independent −1 deletions in *gtr6* that we report here. Both of these −1 deletions occur in strings of “A” residues, which may suggest that these regions are more prone to strand slippage during replication and may be occurring at low levels in a population. Hence, these frameshifts are likely maintained in subsequent generations under appropriate selective pressure and without selection may be reversible. Phenotypic reversion complicates treatment using phage; therefore, further work will be needed to establish whether these mutations are phase variable.

Assessment of the *gtr6*^–^ phenotype in constructed isogenic mutants of KL3- and KL22-carrying strains in a previous study revealed that the absence of the d-Glc*p*NAc side branch results in inhibition of phagocytosis, leading to increased lethality *in vivo* (25). Mutations in the *gtr6* gene (via both frameshifts and insertion sequence [IS] disruption) therefore lead to changes in not only susceptibility to phage but also resistance to the host immune response. Determination of whether mutations that inactivate Gtr6 are globally disseminated and/or are associated with the most resistant isolates belonging to major antibiotic-resistant clonal lineages is therefore important. However, while the disruption of *gtr6* significantly increases the virulence of A. baumannii strains, APK37.1 could be an appropriate choice for therapy or decontamination of clinical environments where outbreaks of highly resistant and hypervirulent *gtr6*^–^ strains are occurring.

Detection of small differences in KL sequences is likely to prove important if other phage isolated for use in phage therapy are also sensitive to the presence of this CPS side chain. However, while Kaptive is an effective tool to detect and type A. baumannii KL sequences, the genes reported as present are based on the detection of ≥90% of the expected translated amino acid sequence (using default parameters). Hence, as the −1 deletion in the AB5001 *gtr6* occurs at the beginning of the gene (Fig. 1B), the translated product meets the minimum 90% coverage threshold, and Kaptive reports the gene as present. Likewise, for HUMC1, the insertion of IS*Aba13* occurs close to the 3′ end of *gtr6* (Fig. 1B), and *gtr6* is again recorded as present given that the translated product (to an alternate stop codon) meets the minimum coverage threshold. Manual inspection of the *gtr6* gene is therefore currently required to identify all disruptions that may abolish Gtr6 function, and rapid automated detection of Gtr6 loss will warrant more sensitive methods.

Interestingly, other APK37.1-sensitive isolates produce K37 (Fig. 2C) or K116 (Fig. 2D) CPSs that do not include a side branch residue attached to position 6 of the terminal d-Gal*p* residue in the main chain (38). The arrangements of the main chain residues are also closely related to the K3-v1 structure as well as the glycosidic linkages between the oligosaccharide K-units that are formed by the Wzy polymerase. While specific sites of CPS cleavage for DpoAPK37.1 were not determined in this study, it is clear that the absence of the d-Glс*p*NAc side branch is necessary for susceptibility to infection by phage APK37.1, and the purified depolymerase digests K3-v1 CPS but not K3 (O. Timoshina and M. Shneider, unpublished observations). The depolymerase may hydrolyze a common structural feature shared by K3-v1, K37, and K116. The ability of APK37.1 to infect strains producing three different CPS types allows for broader application, although further work will be needed to explore phage specificity, host interactions, and therapeutic safety.

## MATERIALS AND METHODS

### Bacterial strains and cultivation.

A. baumannii isolates used in this study (Table 1) were obtained from Mikhail Edelstein (Institute of Antimicrobial Chemotherapy, Smolensk State Medical University, Smolensk, Russia), Bin Liu (TEDA Institute of Biological Sciences and Biotechnology, Nankai University, TEDA, Tianjin, People’s Republic of China), Alexandr Nemec (Laboratory of Bacterial Genetics, National Institute of Public Health, Prague, Czech Republic), Raffaele Zarrilli (Department of Molecular Medicine and Medical Biotechnology, University of Naples Federico II, Naples, Italy), Dean Scholl (AvidBiotics Corp., South San Francisco, CA, USA) and Ry Young (Center for Phage Technology, Texas A&M AgriLife Research and Texas A&M University, College Station, TX, USA). Bacteria were routinely cultivated in 2TY medium at 37°C.

### Bioinformatic analysis of bacterial genomes.

Draft genome sequences of A. baumannii strains (sequenced in this study or downloaded from accession numbers listed in Table 1) were compiled for local analyses. For A. baumannii isolates for which a genome assembly was not already available, genomic material was extracted and sequenced on a MiSeq platform using a Nextera DNA library preparation kit (Illumina, San Diego, CA). Resulting short-read data were assembled into contigs using SPAdes v 3.10 (39). Multi locus sequence typing (MLST) was performed by submitting genome assemblies to the PubMLST database available at https://pubmlst.org/organisms/acinetobacter-baumannii. Resistance determinants were detected using ResFinder v. 4.1 (https://github.com/cadms/resfinder) (40). Sequences of CPS biosynthesis gene clusters were identified in genome assemblies using command-line Kaptive v 2.0.4 (41) with the most recent iteration of the A. baumannii KL reference database that includes 241 KL (11). Sequences of the *gtr6* gene from each genome were extracted, and sequence differences were identified by a pairwise sequence alignment to *gtr6* from ATCC 17978 (GenBank accession number CP012004.1) using CLUSTAL Omega (https://www.ebi.ac.uk/Tools/msa/clustalo/).

### Phage isolation, propagation, and sequencing.

A sewage sample collected in 2018 from the Moscow region, Russia, was used to isolate phage APK37.1 on bacterial lawns of A. baumannii isolates AB5001 and KZ-1101 using methods described previously (42). Phage propagation and purification were executed according to the protocols described earlier (9). Genomic material from APK37.1 was isolated from a purified high-titer stock and sequenced as described previously (9). Briefly, ORFs in the sequenced genome of APK37.1 were predicted using GeneMarkS 4.3, manually checked for fidelity, and annotated according to the conventional gp nomenclature system and submitted to GenBank under accession number MZ967493.1. The positions and lengths of terminal repeats were predicted by searching a region of double read depth in comparison with average read depth across the whole genome.

### Bioinformatic analysis of phage genomes.

Average nucleotide identity (ANI) for comparison of phage genomes was computed using the OrthoANIu tool (43) and USEARCH over BLAST with default settings. Alignment of APK37.1 and Fri1 was visualized using EasyFig software (44). Nucleotide sequences (FASTA) of phage genomes (listed in Table S1 in the supplemental material) were used to construct a proteomic tree using ViPTree (45). The resulting phylogeny was visualized and annotated using the iTOL web interface (https://itol.embl.de/). Percent identity matrices and multiple pairwise alignments of depolymerase sequences were constructed using CLUSTAL Omega with MView (https://www.ebi.ac.uk/Tools/msa/mview/).

### Isolation of the CPSs from A. baumannii isolate AB5001.

Cells of the APK37.1-susceptible A. baumannii isolate AB5001 were harvested by centrifugation (10,000 × *g*, 20 min), washed with phosphate-buffered saline, suspended in aqueous 70% acetone, precipitated, and dried. Dried cell mass (2.88 g) was treated with 45% aqueous solution of phenol (68°C, 1 h) (35), and the extract was dialyzed without layer separation and freed from insoluble contaminations by centrifugation. The resultant solution was concentrated and treated with cold aqueous 50% CCl_3_CO_2_H at 0°C for 1 h. After centrifugation, the supernatant was dialyzed against distilled water. A sample of the native CPS (300 mg) was hydrolyzed with 2% CH_3_CO_2_H (100°C, 2 h). Fractionation of the products was performed by gel permeation chromatography on a column Sephadex G-50 Superfine (53 × 3.5 cm; Amersham Biosciences, Sweden) and gave purified CPS (41.7 mg). Elution was performed with 0.1% acetic acid (HOAc) and monitored using a UV detector (Uvicord, Sweden) at 206 nm.

### Chemical analyses.

A CPS sample (1 mg) of AB5001 was hydrolyzed with 2 M CF_3_CO_2_H (120°C, 2 h), reduced with NaBH_4_ in 1 M NH_4_OH (05 mL, 10 mg/mL, 20°C, 1 h), and acetylated with a mixture of pyridine and acetic anhydride (Ac_2_O) at a ratio of 1:1 (120°C, 2 h). Monosaccharides were analyzed by gas-liquid chromatography (GLC) of the alditol acetates on a Maestro (Agilent 7820) chromatograph (Interlab, Russia) equipped with an HP-5 column (0.32 mm · 30 m) using a temperature program of 160°C (1 min) to 290°C at 7°C min^−1^.

### *O*-Deacetylation of CPS.

A sample of the CPS from strain AB5001 (46 mg) was heated with aqueous 12% NH_4_OH (2 mL) at 38°C for 12 h. Fractionation of the products was performed by gel-permeation chromatography on a column Sephadex G-50 Superfine (53 × 3.5 cm; Amersham Biosciences, Sweden) using 0.1% HOAc for elution and was monitored with a UV detector (Uvicord, Sweden) at 206 nm to give an MPS sample (46 mg).

### Smith degradation.

A sample of the CPS from strain AB5001 (20 mg) was oxidized with aqueous 0.05 M NaIO_4_ (2.7 mL) at 20°C for 40 h in the dark and reduced with NaBH_4_ (80 mg) at 20°C for 16 h. The excess NaBH_4_ was destroyed with concentrated HOAc, the solution was evaporated, methanol was added to the residues (3 × 1 mL) and evaporated, and the residue was dissolved in 0.3 mL of water and applied to a column (108 × 1.2 cm) of TSK HW-40. A modified polysaccharide was eluted with aqueous 0.1% HOAc and hydrolyzed with 2% CH_3_CO_2_H (100°C, 2 h). Fractionation of the products by gel-permeation chromatography on a column (108 × 1.2 cm) of TSK HW-40 in water gave an oligosaccharide (10 mg).

### NMR spectroscopy.

Samples were deuterium exchanged by freeze-drying from 99.9% D_2_O and then examined as solutions in 99.95% D_2_O. NMR spectra were recorded on a Bruker Avance II 600 MHz spectrometer (Germany) at 60°C. Sodium 3-trimethylsilylpropanoate-2,2,3,3-d_4_ (δ_H_ 0, δ_C_ −1.6) was used as an internal reference for calibration. Two-dimensional NMR spectra were obtained using standard Bruker software, and the Bruker TopSpin 2.1 program was used to acquire and process the NMR data. A 60-ms MLEV-17 spin-lock time and a 150-ms mixing time were used in ^1^H,^1^H TOCSY and ROESY experiments, respectively. A 60-ms delay was used for evolution of long-range couplings to optimize ^1^H,^13^C HMBC experiments for the coupling constant *J*_H,C_ of 8 Hz. The following correlations were observed in the ^1^Н,^1^Н ROESY spectrum of the CPS: H-1 of unit **B** with H-3 of unit **C**, H-1 of unit **C** with H-3 of unit **A**, H-1 of unit **A** with H-6 of unit **D**, and H-1 of units **D** and **D′** with H-4 of unit **A**.

### Data availability.

Whole-genome sequence data from this study are deposited in NCBI under accession numbers MZ967493.1, JAPYKX000000000.1, JAPQKD000000000.1, JAPQKA000000000.1, JAPQKC000000000.1, and JANSJS000000000.1.
