# Supplementary material for: Loss of a Branch Sugar in the Acinetobacter baumannii K3-Type Capsular Polysaccharide Due To Frameshifts in the gtr6 Glycosyltransferase Gene Leads To Susceptibility To Phage APK37.1
Source: Microbiol Spectr. 2023 Jan 18;11(1):e03631-22. doi: 10.1128/spectrum.03631-22 (PMC9927144; doi:10.1128/spectrum.03631-22)
Supplement: Supplemental file 1 — Supplemental material. Download spectrum.03631-22-s0001.pdf, PDF file, 0.8 MB [file spectrum.03631-22-s0001.pdf]

Supplementary Information

Table S1. NCBI accession numbers for maximum likelihood phylogeny

Phage	NCBI accession number
APK37.1	MZ967493.1
vB_AbaP_APK81	MT741944.1
vB_AbaP_APK2	MK257719.1
vB_AbaP_APK2-2	MK257720.1
vB_AbaP_APK93	MK257721.1
IME200	NC_028987.2
vB_AbaP_APK37	MK257723.1
AbTP3phi1	OL770263.1
APK15	MZ936315.1
vB_AbaA_fBenAci003	MW056503.1
vB_AbaP_46-62_Aci07	NC_048076.1
APK09	MZ868724.1
vB_AbaP_APK89	MN651570.1
vB_AbaA_fBenAci002	MW056502.1
vB_AbaP_B09_Aci08	NC_048081.1
vB_AbaP_AS12	NC_041914.1
vB_AbaP_APK128	MW459163.1
APK16	MZ868725.1
vB_AbaP_APK48-3	MN614471.1
vB_AbaP_APK116	MN807295.1
vB_AbaP_APK26	MW345241.1
vB_AbaP_APK14	MK089780.1
AB_SZ6	ON513429.1
vB_AbaP_APK44	MN604238.1
vB_AbaP_ZHSHW	OM925528.1
vB_ApiP_P1	NC_042006.1
vB_Api_3043-K38	MZ593174.1
vB_ApiP_P2	NC_042007.1
vB_AbaP_APK87	MN604239.1
AbKt21phiIII	NC_048142.1
vB_AbaP_PMK34	MN433707.1
vB_AbaP_APK32	MK257722.1
Pipo	MW366783.1
Paty	MW366784.1
vB_AbaP_AGC01	MT263719.1
APK20	MZ936316.1
Fri1	KR149290.1
vB_AbaP_PE21	OL964948.1
vB_AbaP_AS11	NC_041915.1
APK77	MZ868726.1
vB_AbaA_fBenAci001	MW056501.1
vB_AbaP_B5	NC_042005.1
APK86	MZ936314.1
SWH-Ab-1	NC_047896.1
vB_AbaP_PD-6A3	KT388102.1
vB_AbaP_B1	NC_042003.1
vB_AbaP_B3	NC_042004.1
vB_AbaP_WU2001	MZ099557.1
phiAB6	KT339321.1
phiAB1	HQ186308.1
vB_AbaP_APK48	MN294712.1
APK127v	ON210142.1
vB_AbaP_IME546	MN061582.1
vB_AbaP_D2	NC_042124.1
WCHABP5	NC_041967.1

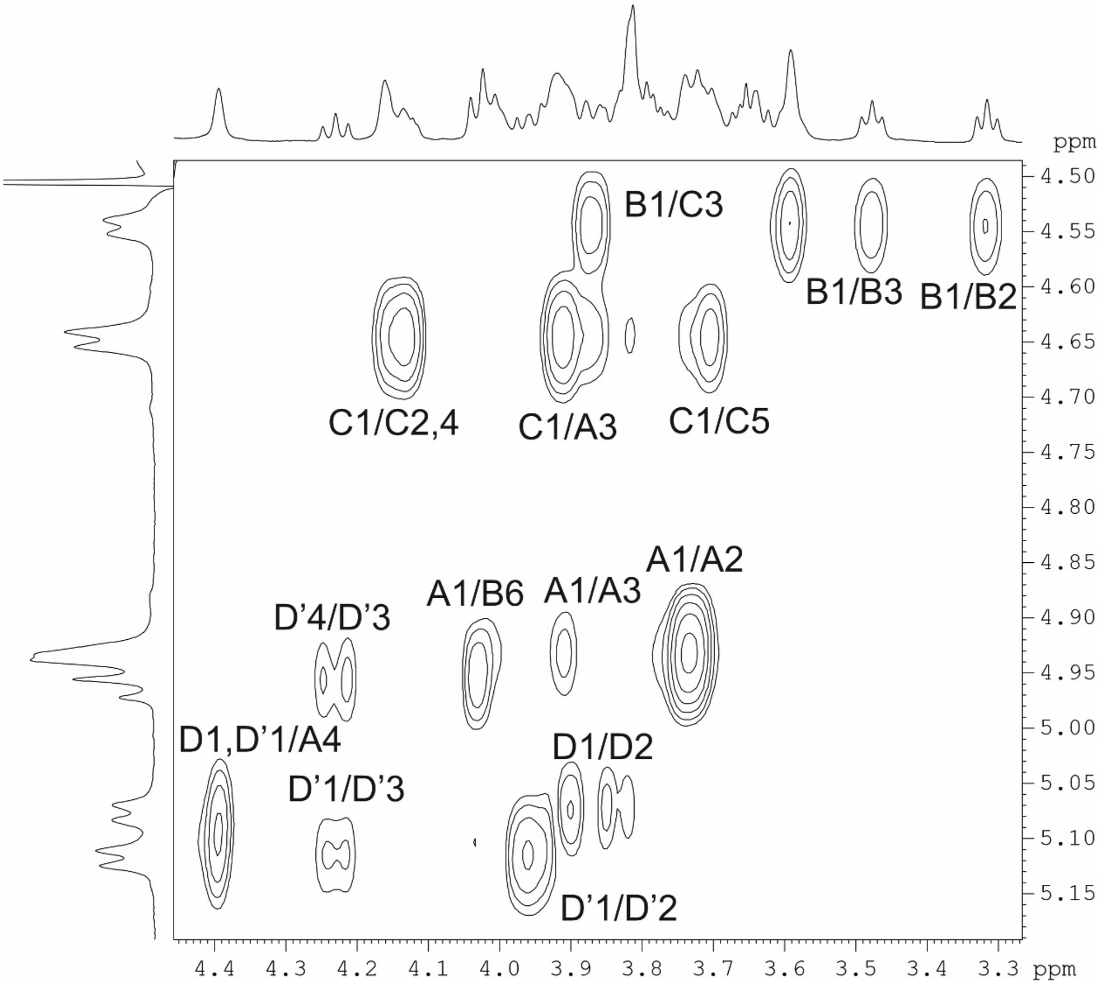

Figure S1. Part of a two-dimensional ^{1}H,^{1}H ROESY spectrum of the AB5001 CPS.

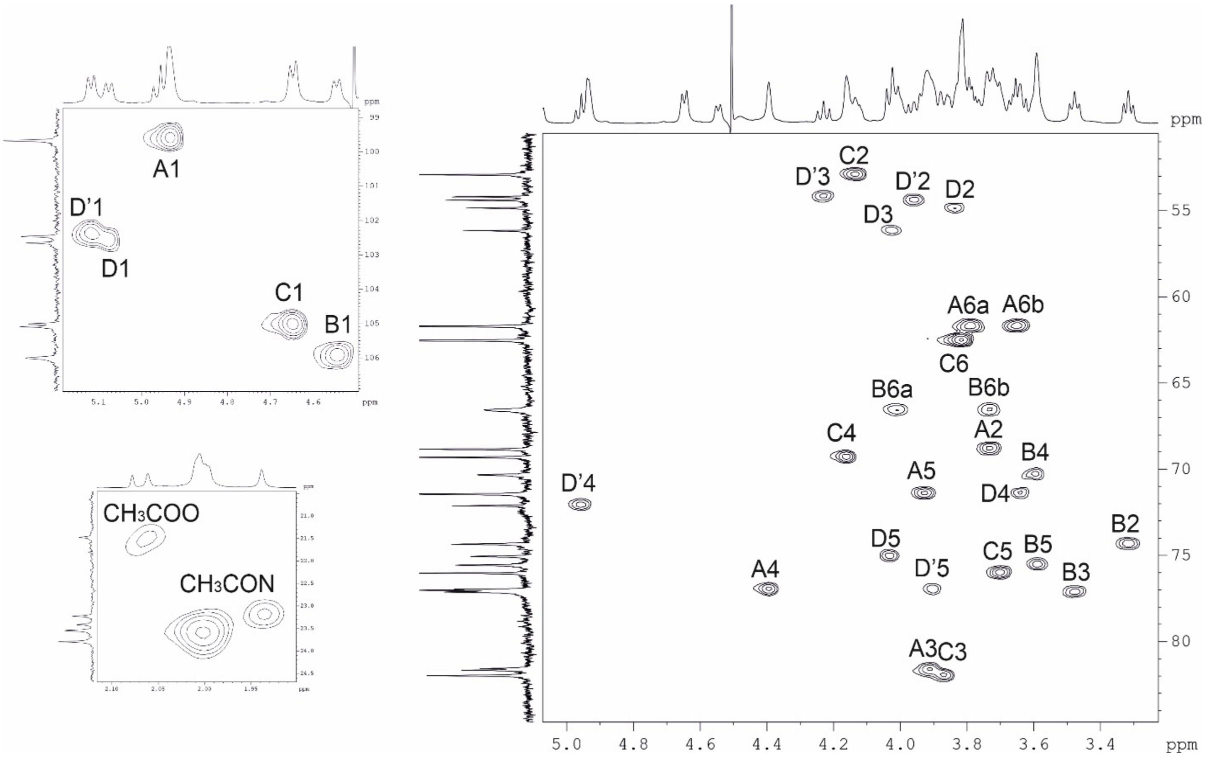

Figure S2. Parts of a two-dimensional $^{1}H,^{13}C$ HSQC spectrum of the AB5001 CPS.

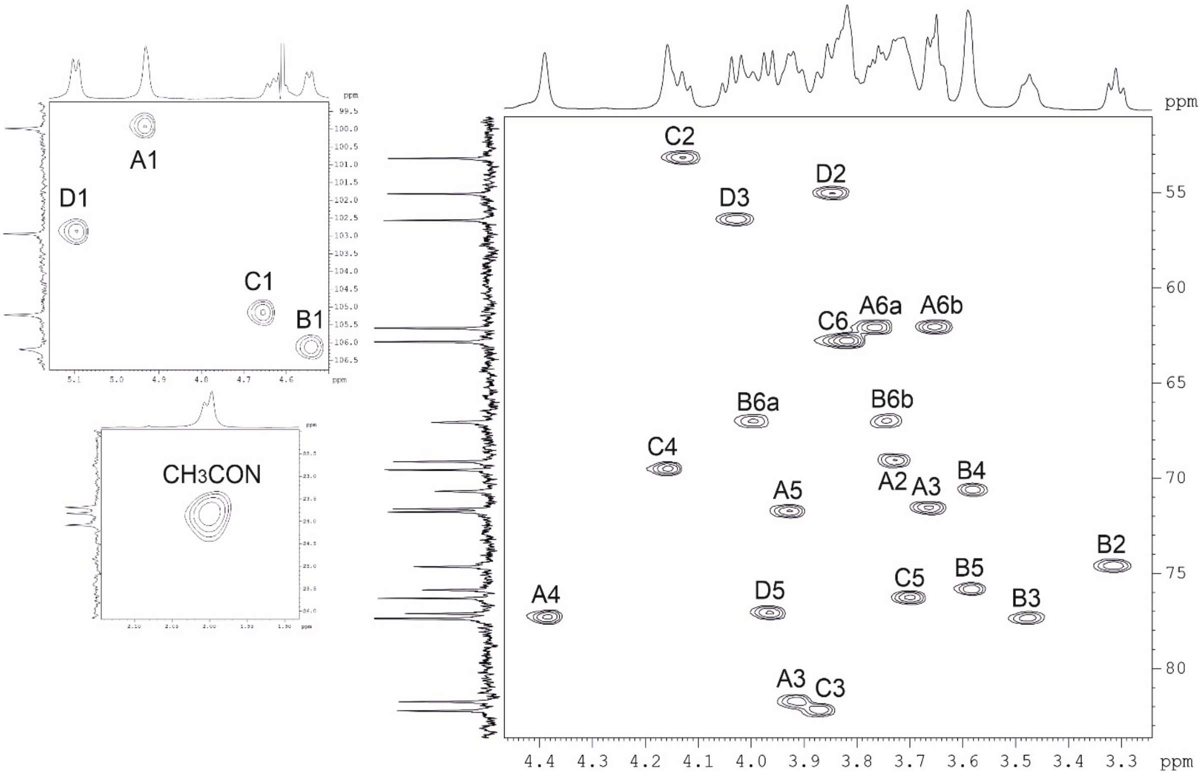

Figure S3. Parts of a two-dimensional $^{1}H,^{13}C$ HSQC spectrum of the MPS of AB5001.

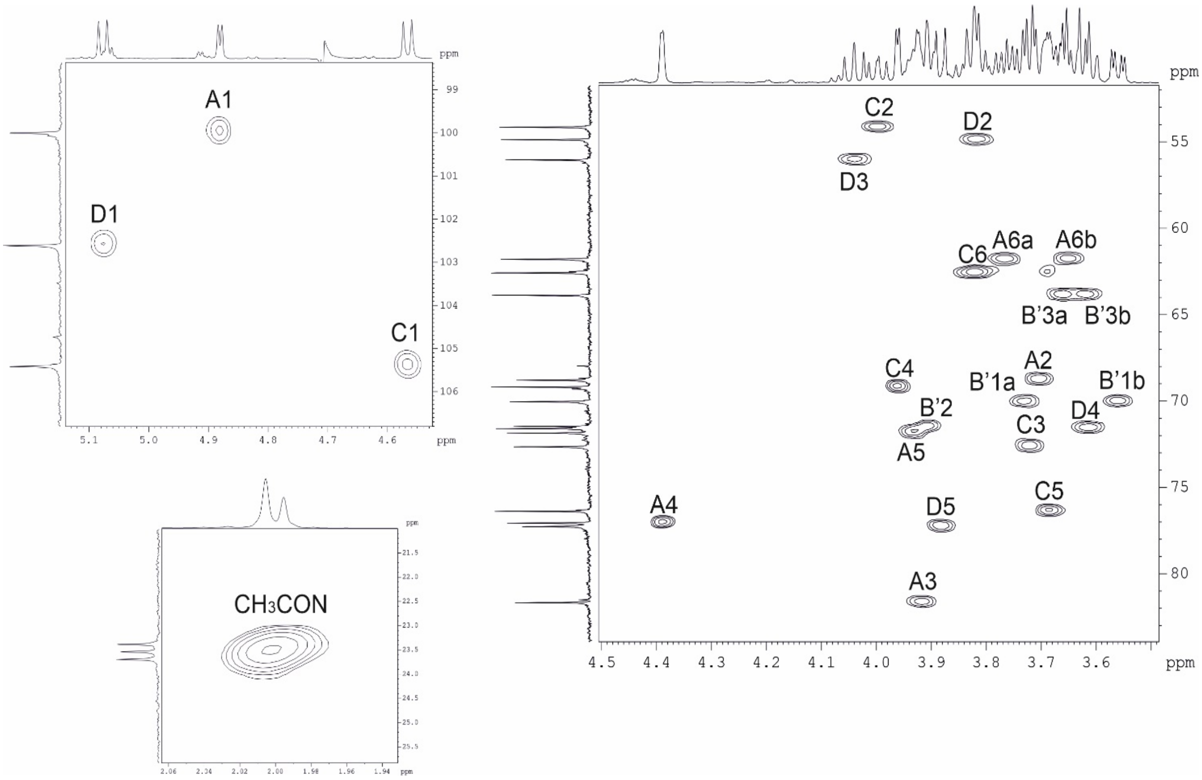

Figure S4. Parts of a two-dimensional ^{1}H,^{13}C HSQC spectrum of the OS derived by Smith degradation of the MPS of AB5001.

```
MAR-13-1378        ATGAAAATTGGATTGACAAAATTTAATTATCCGGAAATTAGATGTGTTACAACTTCGCAG        60
ATCC17978-mff      ATGAAAATTGGATTGACAAAATTTAATTATCCGGAAATTAGATGTGTTACAACTTCGCAG        60
AB5001             ATGAAAATTGGATTGACAAAATTTAATTATCCGGAAATTAGATGTGTTACAACTTCGCAG        60
                   ************************************************************

MAR-13-1378        GAAAATGACTATATAAATTTAAAAAAAATATAATATTTATTATTATTTTAATAATATACCA        120
ATCC17978-mff      GAAAATGACTATATAAATTTAAAAAAAATATAATATTTATTATTATTTTAATAATATACCA        120
AB5001             GAAAATGACTATATAAATTTAA-AAAATATAATATTTATTATTATTTTAATAATATACCA        119
                   ********************** *************************************

MAR-13-1378        GTCATTAAGAATTATTTTCATCGTTTTTTGTTTAAACCTATTTCTGTAAAAAATGTAGAT        180
ATCC17978-mff      GTCATTAAGAATTATTTTCATCGTTTTTTGTTTAAACCTATTTCTGTAAAAAATGTAGAT        180
AB5001             GTCATTAAGAATTATTTTCATCGTTTTTTGTTTAAACCTATTTCTGTAAAAAATGTAGAT        179
                   ************************************************************

MAR-13-1378        GTAATACATTCTTTTAATGATATTTGTTTAACAAATAATAAATGGGTGGTTACTTTCGAG        240
ATCC17978-mff      GTAATACATTCTTTTAATGATATTTGTTTAACAAATAATAAATGGGTGGTTACTTTCGAG        240
AB5001             GTAATACATTCTTTTAATGATATTTGTTTAACAAATAATAAATGGGTGGTTACTTTCGAG        239
                   ************************************************************

MAR-13-1378        ACAATGTTACCACGGTTTTTGGATATTTTGAGTAATCATAAGAATTTGAATCCTGAATAT        300
ATCC17978-mff      ACAATGTTACCACGGTTTTTGGATATTTTGAGTAATCATAAGAATTTGAATCCTGAATAT        300
AB5001             ACAATGTTACCACGGTTTTTGGATATTTTGAGTAATCATAAGAATTTGAATCCTGAATAT        299
                   ************************************************************

MAR-13-1378        ATTTATAATGATGAAATAAATAAATATTTGGAAGTTGTTGCAAGGGATAATTGTTTAGGT        360
ATCC17978-mff      ATTTATAATGATGAAATAAATAAATATTTGGAAGTTGTTGCAAGGGATAATTGTTTAGGT        360
AB5001             ATTTATAATGATGAAATAAATAAATATTTGGAAGTTGTTGCAAGGGATAATTGTTTAGGT        359
                   ************************************************************

MAR-13-1378        GTTATTGCATTGTCAAAAAGCGCAAAAAAAAATTCAATCAGATATTCTAAAAGCTTATCCA        420
ATCC17978-mff      GTTATTGCATTGTCAAAAAGCGCAAAAAAAAATTCAATCAGATATTCTAAAAGCTTATCCA        420
AB5001             GTTATTGCATTGTCAAAAAGCGCAAAAAAAAATTCAATCAGATATTCTAAAAGCTTATCCA        419
                   ************************************************************

MAR-13-1378        AAAGTAAGAGATAAGATTGAAAA-AAAATGTTCGTTTTGTACCCTCCTCAAAAAATATAT        479
ATCC17978-mff      AAAGTAAGAGATAAGATTGAAAAAAAAATGTTCGTTTTGTACCCTCCTCAAAAAATATAT        480
AB5001             AAAGTAAGAGATAAGATTGAAAAAAAAATGTTCGTTTTGTACCCTCCTCAAAAAATATAT        479
                   *********************** ************************************

MAR-13-1378        ACTACTCAACATGAGATTGAAGAAAAAGTCTTAAAACCATTAAAATTAATTTTCGTTGGA        539
ATCC17978-mff      ACTACTCAACATGAGATTGAAGAAAAAGTCTTAAAACCATTAAAATTAATTTTCGTTGGA        540
AB5001             ACTACTCAACATGAGATTGAAGAAAAAGTCTTAAAACCATTAAAATTAATTTTCGTTGGA        539
                   ************************************************************

MAR-13-1378        AATGATTTTTATTTAAAGGGTGGTGCAGAATGTATATTAGCGATTAATGAGTTACTTGAA        599
ATCC17978-mff      AATGATTTTTATTTAAAGGGTGGTGCAGAATGTATATTAGCGATTAATGAGTTACTTGAA        600
AB5001             AATGATTTTTATTTAAAGGGTGGTGCAGAATGTATATTAGCGATTAATGAGTTACTTGAA        599
                   ************************************************************

MAR-13-1378        GAGGGGATAATTTCTGAAAATGAAATTATGTTGACAGTTGTAGGAATTTTAAATCGGACC        659
ATCC17978-mff      GAGGGGATAATTTCTGAAAATGAAATTATGTTGACAGTTGTAGGAATTTTAAATCGGACC        660
AB5001             GAGGGGATAATTTCTGAAAATGAAATTATGTTGACAGTTGTAGGAATTTTAAATCGGACC        659
                   ************************************************************

MAR-13-1378        CATAATTATTCCTTTGGTATTTATCAAGATGATTCTGATTTTTCAAAAAATATTAATACC        719
ATCC17978-mff      CATAATTATTCCTTTGGTATTTATCAAGATGATTCTGATTTTTCAAAAAATATTAATACC        720
AB5001             CATAATTATTCCTTTGGTATTTATCAAGATGATTCTGATTTTTCAAAAAATATTAATACC        719
                   ************************************************************

MAR-13-1378        ATAATTATGAACAGAAAAAAATATTAAAATATATTCTAATGTGGACAATAATAAAGTAATT        779
ATCC17978-mff      ATAATTATGAATAGAAAAAAATATTAAAATATATTCTAATGTGGACAATAATAAAGTAATT        780
AB5001             ATAATTATGAACAGAAAAAAATATTAAAATATATTCTAATGTGGACAATAATAAAGTAATT        779
                   *********** ************************************************

MAR-13-1378        GAAATGATTAGAGAGCATCATATAGGCCTTTTACCGACATGGGCAGATACATTTGGATAT        839
ATCC17978-mff      GAAATGATTAGAGAGCATCATATAGGCCTTTTACCGACATGGGCAGATACATTTGGATAT        840
AB5001             GAAATGATTAGAGAGCATCATATAGGCCTTTTACCGACATGGGCAGATACATTTGGATAT        839
                   ************************************************************

MAR-13-1378        TCTGTTTTAGAGTTCCAAGCTTGTGGTTGCCCTGTAATTTCAACGGATGTTAGAGCATTG        899
ATCC17978-mff      TCTGTTTTAGAGTTCCAAGCTTGTGGTTGCCCTGTAATTTCAACGGATGTTAGAGCATTG        900
AB5001             TCTGTTTTAGAGTTCCAAGCTTGTGGTTGCCCTGTAATTTCAACGGATGTTAGAGCATTG        899
                   ************************************************************

MAR-13-1378        TCGGAAATTAATAATAACGATATTGGTTGGTTAATTAATGTTGATAAAAATAAATATGGT        959
ATCC17978-mff      TCGGAAATTAATAATAACGATATTGGTTGGTTAATTAATGTTGATAAAAATAAATATGGT        960
AB5001             TCGGAAATTAATAATAACGATATTGGTTGGTTAATTAATGTTGATAAAAATAAATATGGT        959
                   ************************************************************

MAR-13-1378        GAGATTGTAGTTGATTCTTATTCAAAGAAAGACTTATGCAGAAGAACTATTATTGATCAG        1019
ATCC17978-mff      GAGATTGTAGTTGATTCTTATTCAAAGAAAGACTTATGCAGAAGAACTATTATTGATCAG        1020
AB5001             GAGATTGTAGTTGATTCTTATTCAAAGAAAGACTTATGCAGAAGAACTATTATTGATCAG        1019
                   ************************************************************

MAR-13-1378        TTGAAGAAGCATATTCTCAGTGCATATGAAAATCCTAATGTTGTTATTAATAAAGGTGTT        1079
ATCC17978-mff      TTGAAGAAGCATATTCTCAGTGCATATGAAAATCCTAATGTTGTTATTAATAAAGGTGTT        1080
AB5001             TTGAAGAAGCATATTCTCAGTGCATATGAAAATCCTAATGTTGTTATTAATAAAGGTGTT        1079
                   ************************************************************

MAR-13-1378        GAATCATTGAATCGTATTAAAAAAGAACATTCTATTGATTATTATAATGATAAAATTAAG        1139
ATCC17978-mff      GAATCATTGAATCGTATTAAAAAAGAACATTCTATTGATTATTATAATGATAAAATTAAG        1140
AB5001             GAATCATTGAATCGTATTAAAAAAGAACATTCTATTGATTATTATAATGATAAAATTAAG        1139
                   ************************************************************

MAR-13-1378        AGTGTTTATACTATTGGTATTTAA        1163
ATCC17978-mff      AGTGTTTATACTATTGGTATTTAA        1164
AB5001             AGTGTTTATACTATTGGTATTTAA        1163
                   ************************
```

Figure S5. Pairwise sequence alignment of *gtr6* from ATCC17978 (NCBI accession number CP012004.1:c3777044-3775881), AB5001 (this study), and MAR-13-1378 (this study). Base deletions are highlighted grey.
